# Real-life introduction of powered circular stapler for esophagogastric anastomosis: cohort and propensity matched score study

**DOI:** 10.1093/dote/doac073

**Published:** 2022-10-11

**Authors:** Stijn Vanstraelen, Willy Coosemans, Lieven Depypere, Yannick Mandeville, Johnny Moons, Hans Van Veer, Philippe Nafteux

**Affiliations:** Department of Thoracic Surgery, University Hospitals Leuven, Leuven, Belgium; Department of Chronic Disease, Metabolism, and Aging, KU Leuven, Leuven, Belgium; Department of Thoracic Surgery, University Hospitals Leuven, Leuven, Belgium; Department of Chronic Disease, Metabolism, and Aging, KU Leuven, Leuven, Belgium; Department of Thoracic Surgery, University Hospitals Leuven, Leuven, Belgium; Department of Chronic Disease, Metabolism, and Aging, KU Leuven, Leuven, Belgium; Department of Thoracic Surgery, University Hospitals Leuven, Leuven, Belgium; Department of Thoracic Surgery, University Hospitals Leuven, Leuven, Belgium; Department of Chronic Disease, Metabolism, and Aging, KU Leuven, Leuven, Belgium; Department of Thoracic Surgery, University Hospitals Leuven, Leuven, Belgium; Department of Chronic Disease, Metabolism, and Aging, KU Leuven, Leuven, Belgium; Department of Thoracic Surgery, University Hospitals Leuven, Leuven, Belgium; Department of Chronic Disease, Metabolism, and Aging, KU Leuven, Leuven, Belgium

**Keywords:** anastomotic leakage, esophageal cancer, esophagectomy, powered circular stapler

## Abstract

Anastomotic leakage after esophagectomy is one of the most feared complications, which results in increased morbidity and mortality. Our aim was to evaluate the impact of a powered circular stapler on complications after esophagectomy with intrathoracic anastomosis for esophageal cancer. Between May 2019 and July 2021, all consecutive oesophagectomies for cancer with intrathoracic anastomosis in a high-volume center were included in this retrospective study. Surgeons were free to choose either a manual or a powered circular stapler. Preoperative characteristics and postoperative complications were recorded in a prospective database, according to EsoData. Propensity score matching (age, body mass index, Eastern cooperative oncology group (ECOG) performance and neoadjuvant therapy) was conducted to reduce potential confounding. We included 128 patients. Powered and manual circular staplers were used in 62 and 66 patients, respectively. Fewer anastomotic leakages were observed with the powered stapler group (OR = 7.3 (95%CI: 1.58–33.7); [3.2% (*n* = 2) vs 19.7% (*n* = 13), respectively; *p* = 0.004]). After propensity score matching, this remained statistically significant (OR = 8.5 (95%CI: 1.80–40.1); [4.1% (*n* = 2) vs 20.4% (*n* = 10), respectively; *p* = 0.013]). Additionally, anastomotic diameter was significantly higher with the powered stapler (median: 29 mm (63.3%) vs 25 mm (57.1%), respectively; *p* < 0.0001). There was no significant difference in comprehensive complication index (*p* = 0.146). A decreased mean length of stay was observed in the powered stapler group (11.1 vs 18.7 days respectively; *p* = 0.022). Postoperative anastomotic leakage after esophageal resection was significantly reduced after the introduction of the powered circular stapler, consequently resulting in a reduced length of stay. Further evaluation on long-term strictures and quality of life are warranted to support these results.

## INTRODUCTION

Esophagectomy remains the cornerstone of the curative treatment of patients with early and locally advanced esophageal cancer but can bear a high morbidity and mortality rate. Anastomotic leakage is one of its most feared complications with rates of 4.7–41%, depending on the series, which results in increased morbidity, length of stay and even mortality.^1–5^ Especially after intrathoracic anastomosis, leakage can lead to infectious mediastinitis with potential deleterious consequences. In an attempt to decrease the anastomotic leak rate, multiple anastomotic techniques have been used but with inconsistent results as the creation of the perfect anastomosis remains challenging (difficult access and technique, potentially ischemic tissues due to impaired vascularization of the conduit).^6^^,^^7^ Consequently, every esophageal surgeon is embarked in a personal quest to define the best possible anastomotic technique.

For decades, circular stapler devices have been used to facilitate creation and improve integrity of intrathoracic anastomosis. Until recently, all circular staplers were fired under manual grip force. Although they typically provide reliable tissue apposition, the manual compression inherits a potential for suboptimal tissue tension due to variable workload, straining and movements during manual firing. Therefore, powered stapling devices, operated using battery packs, have been introduced, first as linear and subsequently as circular devices.^8–10^ Using a battery driven motorized firing system, those devices compensate for the variable grip force of the surgeon, potentially reducing movements at the distal tip of the stapler to allow for more stable stapler positioning as well as staple line formation. These findings were confirmed in an ex-vivo preclinical model.^9^ Moreover, decrease leak rates were seen in several retrospective series comparing manual and powered circular anastomosis in colorectal surgery.^8^^,^^10^^,^^11^ Therefore, the aim of our study was to objectively evaluate the impact of the introduction of a powered circular stapler on postoperative leakage and complications after esophagectomy for esophageal cancer with intrathoracic anastomosis.

## MATERIAL AND METHODS

### Patient selection and data collection

Between May 2019 and July 2021, all consecutive esophagectomies for esophageal cancer with intrathoracic anastomosis were included in this retrospective cohort study from a prospectively acquired database. Patients requiring salvage surgery or total gastrectomy were excluded. All patients were operated through open left thoracoabdominal approach. The use of either a manual (EEA™ Circular Stapler, Medtronic Inc., Minneapolis, MN, USA) or powered (Echelon Circular™ Powered Stapler, Ethicon Inc., Cincinnati, OH, USA) circular stapler for the anastomosis was left to the surgeon’s discretion. With an incidence of around 10%–15% postoperative leakage we calculated that between 100 and 150 would allow for sufficient events to take place and draw preliminary conclusions from this pilot study. Data were collected for preoperative characteristics and Eastern cooperative oncology group (ECOG) performance status, Charlson comorbidity index, tumor stage, postoperative complications (Clavien–Dindo classification and comprehensive complication index [CCI]), length of stay and 90-day mortality, according to Esophageal Complications Consensus Group (ECCG) systematic scoring of complications.^12^^,^^13^ Postoperative leakage was defined as extravasation of contrast on esophagogram or computed tomography, drainage of per oral intake in the chest tube, or visualization of an anastomotic dehiscence on endoscopy, according to ECCG definitions.^13^ Primary endpoint was a difference in occurrence of postoperative anastomotic leakage. Secondary endpoints were overall complications, length of stay and 90-day mortality.

### Anastomotic technique

All surgeons (*N* = 5) performed the operation and anastomosis in the same way. All included patients were operated through open left thoracoabdominal approach. A gastric conduit, pediculated on the right gastroepiploic artery was created. Dissection of the esophagus was performed to a level between the aortic arch and the inferior pulmonary vein. After placement of an automated purse string (Purstring™ 45, Medtronic Inc., Minneapolis, MN, USA) on the muscular wall, the esophagus is transected and an additional manual purse string is placed involving also the mucosal layer of the esophagus using a Prolène™ 3–0 (Ethicon Inc., Cincinnati, OH, USA). Subsequently, the anvil of the stapler is introduced in the esophagus after de visu size estimation by the surgeon. Introduction of the device into the gastric tube after opening the tip of the tube, determination of the location for the anastomosis (end-to-side) and externalization of the device trocar by perforating the conduit. Connection of the trocar to the anvil is established and visually confirmed after which gradual closing of the stapler takes place until sealed according to devices instructions. When the anvil is correctly in contact with the stapler head, the stapler is fired, retracted and removed after loosening of the stapler head by derotating the screw two full circles. The tip of the nasogastric tube is then manually slid intraluminally over the anastomosis into the gastric conduit. The redundant part of the gastric conduit is resected with a linear stapler. After completion, the anastomosis is further reinforced with matrass sutures Ti-Cron™ 3/0(Covidien, Medtronic Inc., Fridley, MN, USA) from the esophagus to the gastric tube, folding the conduit side of the anastomosis over the stapler line against the esophageal wall. Finally, the excessive pedicular fat is wrapped posteriorly around the anastomosis and fixed to the anastomosis and surrounding pleura.

### Propensity matching and statistical analysis

Patient characteristics were retrieved retrospectively from our prospective database: Age, body mass index (BMI), use of neoadjuvant treatment and ECOG performance score. Patients of the powered group were propensity score matched 1:1 to patients in the manual group using nearest neighbor matching for the covariates listed above.

Univariate and multivariate linear regression models (continuous variables) and logistic regression models (binary variables) were used to determine differences in the incidence of postoperative leakage, Clavien–Dindo complications, length of stay and 90-day mortality after propensity score matching. A *P*-value of <0.05 was set as the threshold for statistical significance. Statistical analyses were performed in MEDCALC (v.20.027).

### Legal and ethics

All procedures performed in this study were conducted in accordance with the 1964 Helsinki declaration (as revised in Edinburgh 2000) and national legislation, as well as approved by the ethical committee of University Hospitals Leuven, Belgium (int. ref: s66533). Informed consent was waived because of the retrospective nature of the analysis and data were handled conform European privacy legislation (GDPR).

## RESULTS

### Patient characteristics

A total of 128 consecutive patients were included in this study. Table 1 shows overall patient and operative characteristics for the overall cohort as well as the propensity score matched cohort (*N* = 98).

**Table 1:** Patient and operative characteristics

	Overall cohort			Propensity score matched cohort		
	Non-powered	Powered	*P*-value	Non-powered	Powered	*P*-value
*N* = (%)	66	62		49	49	
Sex Male Female	51 (77.3%) 15 (22.7%)	53 (85.5%) 9 (14.5%)	0.264	38 (77.6%) 11 (22.4%)	41 (83.7%) 8 (16.3%)	0.610
Age (mean)	65.1	64.9	0.892	65.2	64.2	0.708
BMI (*N*, kg/m^2^) Underweight (<18.5) Normal (18.5–25) Overweight (25–30) Obese (>30)	3 (4.5%) 26 (39.4%) 26 (39.4%) 11 (16.7%)	3 (4.8%) 28 (45.2%) 25 (40.3%) 6 (9.7%)	0.696	1 (2%) 23 (46.9%) 19 (38.8%) 6 (12.2%)	1 (2%) 23 (46.9%) 19 (38.8%) 6 (12.2%)	1
Performance status ECOG 0 ECOG 1 ECOG 2 ECOG 3	31 (47.0%) 28 (42.4%) 5 (7.6%) 3 (4.5%)	22 (35.5%) 35 (56.5%) 4 (6.5%) 1 (1.6%)	0.453	21 (42.9%) 25 (51.0%) 3 (6.1%) 0 (0.0%)	21 (42.9%) 25 (51.0%) 3 (6.1%) 0 (0.0%)	1
Charlson Comorbidity Index 0 1 2 3 4 5 6 7	21 (31.8%) 14 (21.2%) 16 (24.2%) 9 (13.6%) 2 (3.0%) 1 (1.5%) 2 (3.0%) 1 (1.5%)	25 (40.3%) 13 (21.0%) 14 (22.6%) 5 (8.1%) 2 (3.2%) 1 (1.6%) 2 (3.2%) 0 (0.0%)	0.924	16 (32.7%) 12 (24.5%) 11 (22.4%) 5 (10.2%) 2 (4.1%) 0 (0.0%) 2 (4.1%) 1 (2.0%)	20 (40.8%) 12 (24.5%) 12 (24.5%) 4 (8.2%) 0 (0.0%) 0 (0.0%) 1 (2.0%) 0 (0.0%)	0.610
Treatment Post-induction Primary surgery	55 (83.3%) 11 (16.7%)	50 (80.6%) 12 (19.4%)	0.819	43 (87.8%) 6 (12.2%)	43 (87.8%) 6 (12.2%)	1
Surgeon Surgeon 1 Surgeon 2 Surgeon 3 Surgeon 4 Surgeon 5 (consultant)	22 11 19 9 5	12 7 6 35 2	NA	16 7 15 6 5	6 7 6 18 2	NA
Stapler size (mm) 21 25 28 29 31	2 (3.0%) 38 (57.6%) 26 (39.4%) / /	/ 19 (30.6%) / 42 (67.7%) 1 (1.6%)	**<0.0001**	2 (4.1%) 28 (57.1%) 19 (38.8%) / /	/ 17 (34.7%) / 31 (63.3%) 1 (2.0%)	**<0.001**

BMI, Body mass index; ECOG, Eastern Cooperative Oncology Group.

Statistical significant difference as *p* <0.05.

### Overall cohort

In the overall cohort, no difference in demographics characteristics was seen between both groups. However, the size of the anastomosis however was significantly larger in the powered group compared to the manual group (median diameter of 29 mm vs 25 mm, respectively, *P* < 0.0001).

### Propensity score matched cohort

Patients were matched according to age, ECOG performance, BMI and neoadjuvant treatment (Table 1). The majority of patients were male (80%), ECOG 0 or 1 (>90%) and underwent neoadjuvant therapy (87.8%). The size of the anastomosis was significantly larger in the powered cohort group with a median of 29 mm and 25 mm in the powered and non-powered group (*P* < 0.001), respectively.

### Postoperative leakage and complications

Table 2 summarizes the postoperative complications and general outcome after esophagectomy in both the overall cohort and propensity matched cohort.

**Table 2:** Postoperative complications and outcome

	Overall cohort			Propensity score matched cohort		
	Non-powered	Powered	*P*-value	Non-powered	Powered	*P*-value
*N* = (%)	66	62		49	49	
Anastomotic leak	13 (19.7%)	2 (3.2%)	**0.004**	10 (20.4%)	2 (4.1%)	**0.013**
Clavien-Dindo Grade 0 Grade 1 Grade 2 Grade 3A Grade 3B Grade 4 Grade 5	14 (21.2%) 1 (1.5%) 26 (39.4%) 4 (6.1%) 5 (7.6%) 15 (22.7%) 1 (1.5%)	19 (30.6%) 4 (6.5%) 20 (32.3%) 5 (8.1%) 4 (6.5%) 10 (16.1%) 0 (0.0%)	0.488	12 (24.5%) 0 (0.0%) 21 (42.9%) 2 (4.1%) 4 (8.2%) 9 (18.4%) 1 (2.0%)	13 (26.5%) 1 (2.0%) 17 (34.7%) 5 (10.2%) 4 (8.2%) 9 (18.4%) 0 (0.0%)	0.711
Comprehensive Complication Index (mean)	33.8	23.0	**0.005**	33.0	25.9	0.146
Length of stay (mean)	18.3	11.9	**0.026**	18.7	11.1	**0.022**
90-day mortality	4 (6.1%)	1 (1.6%)	0.366	3 (6.1%)	1 (2.0%)	0.312

### Overall cohort

In the full cohort, an anastomotic leakage rate of 19.7% (*n* = 13) was noted in the manual group compared to 3.2% (*n* = 2) in the powered group (*P* = 0.004). This corresponds to a relative risk reduction of 83% and an odds ratio (OR) of 7.3 (95% confidence interval [CI]: 1.58–33.74). Table 2 shows the complete Clavien–Dindo complication distribution. There was no significant difference between both groups for the individual Clavien–Dindo scales (*P* = 0.488). The comprehensive complication index was significantly higher in the manual group (mean: 33.8 vs 23.0, respectively; *P* = 0.005). Mean length of stay was also significantly longer in the manual group (11.9 days vs 18.3 days, respectively; *P* = 0.026). However, there was no significant difference in 90-day mortality between the two groups (manual: 6.1% [*n* = 4] vs powered: 1.6% [*n* = 1]; *P* = 0.366). Mortality was not related to the presence of an anastomotic leakage.

### Propensity score matched cohort

After propensity score matching anastomotic leakage rates remained significantly lower in the powered compared to the manual group respectively (4.1% [*n* = 2] and 20.4% [*n* = 10], respectively; *P* = 0.013), corresponding with a relative risk reduction of 80% and OR of 8.5 (95%CI: 1.80–40.1). All leaks were observed after induction therapy, however, there was no statistically significant difference between chemotherapy (CTx) alone or chemoradiotherapy (CRTx) (powered-group: CTx 0/30 vs CRTx 2/19; *P* = 0.072 non-powered: 5/24 vs 5/25; *P* = 0.950). Clavien–Dindo complications and comprehensive complication index were comparable between both groups after propensity score matching (*P* = 0.711 and *P* = 0.146, respectively). Mean length of stay remained significantly lower in the powered group (11.1 days vs 18.7 days, *P* = 0.022). Finally, 90-day mortality was not significantly different between the groups (*n* = 1 [2.0%] in the powered group and *n* = 3 [6.1%] in the manual group; *P* = 0.312). Mortality was not related to the presence of an anastomotic leakage.

## DISCUSSION

In this retrospective series, the use of a powered circular stapling device, for creation of anastomosis, decreases the rate of anastomotic leakage significantly when compared to manual circular staplers after open esophagectomy with intrathoracic anastomosis after performing a propensity score matched analysis.

Several risk factors have been associated with anastomotic leakage. Hagens *et al*.^5^ identified a high body mass index, diabetes mellitus and chronic obstructive pulmonary disease as risk factors for anastomotic leakage. Influence of total radiation dose on the gastric conduit and especially cardiovascular disease on postoperative leakage have also been described.^14–18^ Therefore, we used a propensity score matching to correct for BMI and related diabetes mellitus, ECOG performance index as a surrogate for both cardiovascular and pulmonary functioning, and neoadjuvant therapy in order to create two groups equally at risk for the development of anastomotic leakage. We could observe a significant 80% reduction in anastomotic leakage between the two groups in favor of the powered circular stapler (OR = 8.5 (95%CI: 1.80–40.1); [4.1% (*n* = 2) vs 20.4% (*n* = 10), respectively; *P* = 0.013]).

Powered stapling devices have been designed to create a more uniform stapler closure combined with a more evenly distributed compression during firing as compared to manual stapling devices. Furthermore, powered stapling devices are battery driven compensating for the manual grip force applied during firing with a manual stapler which potentially results in a reduction of distal tip movements during firing. As a consequence, less tension is generated on staples and anastomosis during its creation. All those elements could potentially explain the decrease in anastomotic leak rate in this series. Adding a three stapling technology in upcoming manual staplers as compared to double stapling is unlikely to change the outcomes as Mazaki *et al*.^19^ could find no difference in leakage rates between the two technologies. Additionally, anastomotic diameter is known to be a risk factor for anastomotic leakage; however, this is only noted in diameters of 31 mm and above.^20^^,^^21^ Several other studies in both esophageal and colorectal cancer have shown no statistically significant difference between stapler sizes between 25 and 31 mm.^22–25^ Since only one patient in the powered group was treated with a 31 mm device, this could not explain the difference in postoperative leakage. In our opinion, a bigger overall diameter in the powered group should have increased its susceptibility to anastomotic dehiscence and therefore is a factor against the powered stapler group. Since, on the contrary, even in this case the incidence of anastomotic leakage dropped significantly, it further supports our believes that the powered firing provides the more uniform and solid anastomosis, rather than the amount of staples lines or diameter. A definite conclusion on both can only be made after performing a randomized controlled trial. However, the most important matter is to provide the right size of stapler for the diameter of the esophagus. Over- or undersizing will result in a distorted anastomosis, more prone to anastomotic dehiscence.

Postoperative leakage has been associated with an increased morbidity and even mortality. According to literature, anastomotic leaks result in a two times higher risk for pneumonia, ten times higher risk for empyema, an increased hospital stay of more than 9 days and the reason for readmission in 24.3% of the cases.^14^^,^^18^^,^^26^ Thirty-day mortality rates of up to 11% have been described after an anastomotic leak, leading in 80% of the case to re-intervention.^14^^,^^18^^,^^26^ In our propensity score matched cohort, we could observe the same results in terms of length of stay with a significant difference of 7 days on the mean hospital stay. No difference in mortality was seen as only one death in the manual group was recorded.

Even though, in the unmatched cohort a significant difference in comprehensive complication index was present, this could not be confirmed in the matched cohort. We believe this is at least partially due to early detection of leaks with imaging studies (contrast swallow or CT chest with per oral contrast) day 4 after surgery which allows early supportive treatment with antibiotics, nil by mouth continued drainage through a chest tube and extensive use of endoluminal vacuum therapy in case of a leak. Endoluminal vacuum therapy has shown a success rate of up to 93% with a change interval of 2–3 times a week and is applicable for a wide variety of defects.^6^^,^^27^^,^^28^

A significant reduction in anastomotic dehiscence could also be observed after introduction of a powered circular stapler in colorectal surgery for left sided resections, as well as a reduction in length of stay and 30-day readmission.^8^^,^^11^ Pollack *et al*. calculated an impressive economic benefit of $53,987 annually from the use of a powered circular stapler in left sided resection with a reduction risk of 73%, assuming 100 resections per year (1.8% vs 6.6%, respectively; *P* < 0.0001). This included cost related to anastomotic leakage, prolonged length of stay, readmission and non-home discharges.^11^^,^^29^ Although this decrease in cost was not evaluated in our series, the risk reduction of 80% in our study was even higher suggesting an even bigger economic benefit.

A strength of this trial is that both groups of patients are contemporary and as such we could avoid comparison with historical cohorts. No selection was performed except for rescue surgery. We therefore truly believe that those results are very representative of the current daily situation. This trial has been conducted in a high-volume academic center with all participating surgeons having at least a 5-year experience and performing at least 20 resections per year. Moreover, in this retrospective analysis of an unselected group of patients, a propensity score matching was performed to correct for potential confounders.

Additionally, all anastomoses were performed in a standardized way by all surgeons to avoid introducing other potential confounding factors than the type of stapler. As previously mentioned, techniques known to potentially decrease risk of anastomosis leakage, like reinforcement of the anastomosis by a row of stiches to decrease tension on staplers and omental wrapping of the anastomosis were used in exactly the same way in both groups of patients.^4^^,^^27^^,^^30–32^

This study however presents some weaknesses. The first one is the sample size. Although a total cohort of 128 patients allows for differentiation between the two surgical approaches. It is insufficiently powered to detect minor differences in postoperative complications. Comparative studies with larger samples sizes or, in the future, meta-analyses are necessary to detect these differences.

Secondly, despite the propensity score matched analysis and correction for preoperative confounders, the studied patient cohort remains a population of patients who were operated through an open thoracoabdominal approach. Whether these results can be extrapolated to anastomosis creation during minimally invasive esophagectomy is not known, although we believe that the described potential advantages of a powered stapling devise remain independent of the approach. Further clinical evidence is indeed necessary to confirm this idea.

Additionally, a selection bias might exist since the use of a new device might unintendedly influence the meticulousness by which the anastomosis was performed. On the other hand, surgeons were free to choose their preferred stapler and yet believed in their own choice. We could not observe major differences in the choice of device between the surgeons. Furthermore, at the end of the study, all surgeons started to use the power circular stapler and the preliminary results were confirmed. After 5 months, 23 patients consecutive patients underwent an esophagectomy through left thoracoabdominal approach with a powered stapled intrathoracic anastomosis and only one leak was observed (4.35%).

However, further research on anastomotic stenoses and quality of life is warranted to determine the long-term impact of the powered circular stapler in upper gastrointestinal surgery. It is believed that anastomotic strictures are related to postoperative anastomotic leakage but also to the size of the anastomosis and therefore less frequent with hand sewn types of anastomosis.^16^^,^^33^ Since leak rate was lower and the median size of the anastomosis was significantly higher in the powered group, we assume that in the long-term less strictures will be observed. Further follow up data will have to address these assumptions.

## CONCLUSION

The introduction of a powered circular stapler contributed to an 80% risk reduction of anastomotic leakage after esophageal resection for cancer with intrathoracic anastomoses in a propensity score matched monocentric observational series. This consequently resulted in a reduced length of stay. Further confirmation in larger series and, ideally, a randomized controlled trial and evaluation on long-term strictures and quality of life are warranted to support these results and adjust practice accordingly.

## Conflicts of interest

On demand, the department provides cadaver courses and observerships for Medtronic Inc., Minneapolis, MN, USA and Ethicon Inc., Cincinnati, OH, USA. The authors do not receive individual grants or fees from any company or institution.
