# Correction to: Real-life introduction of powered circular stapler for esophagogastric anastomosis: cohort and propensity matched score study

**DOI:** 10.1093/dote/doac107

**Published:** 2022-12-10

This is a correction to: Stijn Vanstraelen, Willy Coosemans, Lieven Depypere, Yannick Mandeville, Johnny Moons, Hans Van Veer, Philippe Nafteux, Real-life introduction of powered circular stapler for esophagogastric anastomosis: cohort and propensity matched score study, *Diseases of the Esophagus*, 2022;, doac073, https://doi.org/10.1093/dote/doac073

In the originally published version of this manuscript, the following sentence required a correction:

Although the manual stapler was based on double stapling technology and the powered stapler on a tri-stapling technology, Mazaki et al.19 could find no difference in leakage rates between the two technologies.

The sentence has been updated as follows:

Adding a three stapling technology in upcoming manual staplers as compared to double stapling is unlikely to change the outcomes as Mazaki et al.19 could find no difference in leakage rates between the two technologies.

